# Increased Emergency Department Hallway Length of Stay is Associated with Development of Delirium

**DOI:** 10.5811/westjem.2021.1.49320

**Published:** 2021-04-09

**Authors:** Kate van Loveren, Arnav Singla, Liron Sinvani, Christopher Calandrella, Thomas Perera, Martina Brave, Lance Becker, Timmy Li

**Affiliations:** * North Shore University Hospital, Department of Emergency Medicine, Manhasset, New York; † Center for Health Innovations and Outcomes Research, Feinstein Institutes of Medical Research, Northwell Health, Manhasset, New York; ‡ Donald and Barbara Zucker School of Medicine at Hofstra/Northwell, Department of Emergency Medicine, Manhasset, New York; § Donald and Barbara Zucker School of Medicine at Hofstra/Northwell, Department of Medicine, Division of Hospital Medicine, Manhasset, New York

## Abstract

**Introduction:**

Our study aimed to determine 1) the association between time spent in the emergency department (ED) hallway and the development of delirium and 2) the hospital location of delirium development.

**Methods:**

This single-center, retrospective chart review included patients 18+ years old admitted to the hospital after presenting, without baseline cognitive impairment, to the ED in 2018. We identified the Delirium group by the following: key words describing delirium; orders for psychotropics, special observation, and restraints; or documented positive Confusion Assessment Method (CAM) screen. The Control group included patients not meeting delirium criteria. We used a multivariable logistic regression model, while adjusting for confounders, to assess the odds of delirium development associated with percentage of ED LOS spent in the hallway.

**Results:**

A total of 25,156 patients met inclusion criteria with 1920 (7.6%) meeting delirium criteria. Delirium group vs. Control group patients spent a greater percentage of time in the ED hallway (median 50.5% vs 10.8%, P<0.001); had longer ED LOS (median 11.94 vs 8.12 hours, P<0.001); had more ED room transfers (median 5 vs 4, P<0.001); and had longer hospital LOS (median 5.0 vs 4.6 days, P<0.001). Patients more frequently developed delirium in the ED (77.5%) than on inpatient units (22.5%). The relative odds of a patient developing delirium increased by 3.31 times for each percent increase in ED hallway time (95% confidence interval, 2.85, 3.83).

**Conclusion:**

Patients with delirium had more ED hallway exposure, longer ED LOS, and more ED room transfers. Understanding delirium in the ED has substantial implications for improving patient safety.

## INTRODUCTION

Delirium is a common, costly, and devastating condition affecting up to 50% of hospitalized, older patients^1^ and incurs a multibillion dollar financial burden on annual healthcare expenditures.^1^,^2^,^3^ Delirium is a proponent of iatrogenic complications, such as falls, infections, and pressure ulcers, that lead to longer hospital lengths of stay (LOS) and higher rates of hospital discharge to a skilled nursing facility and long-term placement.^4^–^9^ In addition, its development has been shown to increase the risk of mortality by 70% in the first six months after an emergency department (ED) visit.^10^ Because of these known deleterious outcomes, delirium prevention, recognition, and management have been identified as a national priority with regard to patient safety and quality of care.

The rates of delirium development can vary depending on the hospital unit ranging from 6–56% in the general medicine surgery unit,^2^ upwards of 81% in the intensive care unit (ICU),^8^ and 5–17% in the ED.^11^–^14^ Prior literature on environmental risk factors for delirium development has focused on inpatient settings. Environmental factors such as sensory impairment,^15^ sleep interruptions,^11^ and inadequate social interactions with familiar persons^16^ have been identified as risk factors on inpatient floors. However, the environment of the ED has not been adequately investigated with regard to its effect on delirium development.

The environmental risk factors identified on inpatient floors are not only present in the ED but likely amplified. The ED is particularly associated with an uncomfortable, unfamiliar, and disorienting environment. In particular to the ED environment, EDs across the nation often face overcrowding, which necessitates the treatment of patients in non-treatment areas such as the hallway.^17^ Due to inpatient crowding, the boarding of admitted patients in the ED, typically in the hallway, is another source of exhaustion of ED resources.^17^ This trend will appreciate over time as an increasing number of older adults present to the ED.^18^ Persons over the age of 65 are at higher risk of developing delirium,^19^–^21^ which requires an assessment of the impact of the ED’s environment on the development of delirium.

The ED hallway is a unique hospital setting where patients are placed while they are under ED care or while they are admitted and waiting for transfer to a hospital unit. The chaotic setting of the ED hallway may provide constant, uninterrupted exposure to the known environmental risk factors that contribute to the development of delirium. The identification of the ED hallway is a novel site of research to understand its role in the development of delirium. The objectives of this study were to 1) assess the association between time spent in the ED hallway and delirium development; and 2) determine the hospital location of delirium development.

## METHODS

### Study Design and Setting

This was a single-center, retrospective cohort study. The hospital is a 756-bed, academic, quaternary care center, verified as a Level I trauma center, with a yearly ED volume of approximately 90,000 patients. Our health system’s institutional review board determined that this study qualified as a quality improvement project, with a waiver of informed consent.

### Selection of Participants

We identified all patients 18 years or older presenting to the ED between January 1–December 31, 2018, who were subsequently admitted to the hospital. In the hospital studied, there is no standardized delirium screening, which required us to use surrogate markers for identifying delirium development. A preliminary review of the literature as well as of the electronic health record (EHR) was conducted of patients with and without an ED discharge diagnosis of delirium (n = 27 and n = 27, respectively), in order to better understand local EHR delirium documentation and to determine what criteria could best identify delirium development. We used previous studies identifying delirium through retrospective chart reviews as models for the electronic data query,^22^–^25^ and the 54-patient pilot abstraction sought to validate these methods.

**Population Health Research Capsule**

What do we already know about this issue?

*The emergency department (ED) hallway has potential for amplification of known environmental risk factors for the development of delirium*.

What was the research question?

*What is the association between time spent in the ED hallway and the development of delirium?*

What was the major finding of the study?

*Patients are 3.31 times more likely to develop delirium with each percent increase of time spent in the ED hallway*.

How does this improve population health?

*The findings emphasize the need to expand delirium prevention and management in the ED*.

The 54 patients selected for this pilot abstraction were randomly chosen based off of all patients presenting to the ED within approximately the previous two years (January 1, 2017–October 30, 2018) at the time the pilot was conducted. Patients who presented to the ED with an ED diagnosis of delirium (n = 180) were considered part of the Delirium group for the pilot; patients with all other ED diagnoses were part of the Control group. Twenty-seven patients (15%) of this Delirium group were randomly chosen, and 27 Control patients were randomly selected. After reviewing the EHR records of 54 patients, including all nursing and physician documentation and provider orders, we determined delirium development based on any provider order for delirium management, including pharmacologic agents, observation orders, and Confusion Assessment Method (CAM) documentation, which was corroborated by provider documentation of frequently used key words identified from prior literature^22^–^25^ to describe delirium symptoms (Table 1).

We omitted from the Delirium group those patients with orders for delirium management and documentation of delirium symptoms who also had dementia (indicated by orders for donepezil or documentation of dementia); a stroke (indicated by orders for clopidogrel); patients who were on a 1:1 observation; or who had documentation of certain key words indicating altered cognition (Table 1). Based on the pilot abstraction, these patients shared in common provider orders for delirium management and documentation of key words describing delirium symptoms with Delirium group patients but did not have delirium. Patients excluded from the Delirium group were also excluded from the Control group.

To assess delirium development during the hospital stay, we excluded patients if they presented to the ED with baseline altered cognition, such as a chief complaint of intoxication, alcohol or drug withdrawal, altered mental status, suicidal ideations, or psychological conditions such as delusions, psychiatric evaluations, dementia, or delirium. These patients were excluded from both the Delirium and Control groups. The Control group included all other patients 18 years or older who did not meet our criteria for delirium and were admitted via the ED in 2018. We based the final data query for this study on methods from prior literature,^22^–^25^ including methods performed at the site of this study,^24^,^25^ which were validated by a pilot abstraction in order to represent local practices for delirium management and documentation of symptoms.

### Measurements

We performed an electronic data query to extract data from our health system’s EHR. Variables obtained from the electronic data query included patient demographics, all timestamps from ED arrival to inpatient discharge, ED bed locations and timestamps, Charlson Comorbidity Index (CCI), chief complaint, and admission and discharge diagnoses. We reviewed the timestamps for orders for medications and observations and for documentation of positive CAM screening to use as surrogate markers of delirium to determine at what point delirium developed during the patient’s hospital course.

### Outcomes

The primary outcome variable was the development of delirium. The primary independent variable was the proportion of time spent in the ED hallway, “% ED hallway time.” We calculated percent ED hallway time by dividing ED hallway LOS (the cumulative time a patient spent in a designated hallway bed location from ED arrival to ED discharge) by ED LOS (defined as the total time spent in the ED between ED arrival and ED discharge). We defined ED room transfers as the total number of times a patient switched bed locations in the ED during their ED LOS. Hospital LOS, which includes ED LOS, was equivalent to the time a patient spent from ED arrival to inpatient/hospital discharge.

### Analysis

We performed all data analyses using SAS 9.4 (SAS Institute Inc, Cary, NC). Descriptive statistics were used to describe the study sample. We assessed differences in characteristics between Delirium and Control patients using Wilcoxon rank-sum or chi-square tests. To address our first objective, we constructed a multivariable logistic regression model to assess the independent association between proportion of time spent in the ED hallway and development of delirium. We included covariates that are clinically important or statistically significantly different between the two groups of patients at baseline. Age is a clinically significant covariate, and race, CCI, ED LOS, and number of room transfers were significantly different at baseline between the Delirium and Control groups; thus, these covariates were included in the initial multivariable regression model. Covariates were individually removed from the model starting with the covariate with the largest *P*-value. We only retained covariates that were clinically significant and/or statistically significant at the *P*<0.05 level. Adjusted odds ratios, their corresponding 95% confidence intervals (CI), and type 3 *P*-values are presented.

To address our second objective we cross-referenced the time of delirium identification with the patient’s location in the hospital as documented in the patient’s EHR to determine where in the patient’s hospital course delirium was identified. The time of delirium identification was based on the first documented order time for a pharmacologic agent (lorazepam, quetiapine, haloperidol, or valproic acid); order time for special observation (Constant Observation, Enhanced Supervision, Non-Violent Non-Self-Destructive Level 1 Restraint, or Violent Self-Destructive Level 2 Restraint); or first inpatient-nursing documentation of a positive CAM screening,^26^–^29^ a validated screening tool to assess delirium symptoms. If the criteria for delirium identification were met while the patient was in triage, under ED care, or in holding (inpatient boarding in the ED), then a patient was identified with delirium in the ED; if the criteria were met while a patient was admitted into the hospital then the patient was identified with delirium as an inpatient.

## RESULTS

### Characteristics of the Study Subjects

**Figure 1:**
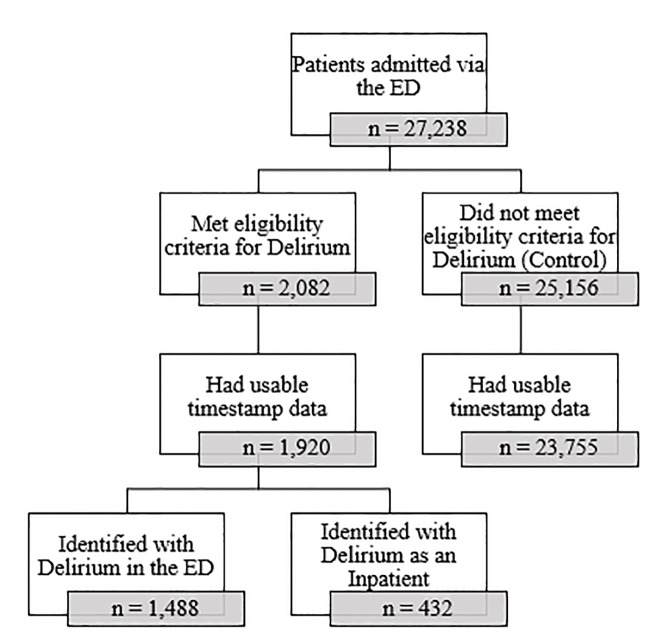
Patient flow diagram for study of association between delirium and emergency department (ED) site of care (data availability January 1–December 31, 2018).

As shown in Figure 1, 27,238 patients presented to the ED and were subsequently admitted to the hospital. Of these patients 2,082 met the eligibility criteria for the Delirium group, and 25,156 met the criteria for the Control group. However, due to missing timestamp data, 162 patients were excluded from the Delirium group and 1,401 were excluded from the Control group; therefore, the analytic sample was comprised of 1,920 patients in the Delirium group and 23,755 patients in the Control group. Of the 1,920 patients in the Delirium group, 1,488 (77.5%) developed delirium in the ED and 432 (22.5%) developed delirium in the inpatient setting.

As shown in Table 2, patient age, gender, and ethnicity were not significantly different between the Delirium and Control groups. However, race and CCI were significantly different between the two groups. There was a higher proportion of White patients in the Delirium group compared with the Control group (68.5% vs 60.4%) and a higher proportion of patients with a CCI of 3 in the Delirium group compared with the Control group (15.1% vs 10.9%).

### Main Results

The time of delirium development was most commonly identified by an order for a pharmacologic agent followed by observation orders and a positive CAM screen completed by inpatient nursing staff (Table 3). Of the patients in the Delirium group, 1,515 (78.9%) received at least one pharmacologic agent, the most frequently ordered medications being lorazepam (n = 907, 47.2%) and quetiapine (n = 322, 16.8%). Among Delirium patients, 397 (20.7%) received at least one special observation order such as “constant observation” (n = 188, 9.8%) and “enhanced supervision” (n = 168, 8.8%). The least frequently used measure of identifying or beginning treatment for delirium was the inpatient CAM screening tool, as only eight (0.4%) patients in the Delirium group received this assessment (Table 3).

**Figure 2:**
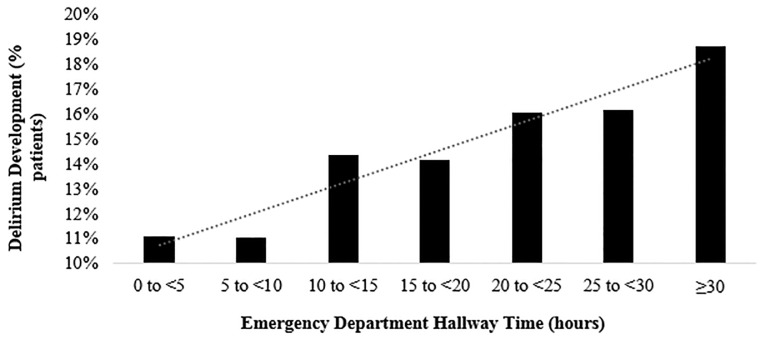
Impact of time spent in the emergency department hallway on the development of delirium.

Patients identified with delirium vs those in the Control group had a greater proportion of their ED stay in the hallway (median of 50.5% vs 10.8%, *P*<0.001) (Table 4). The percentage of patients who developed delirium increased based on the cumulative hours all patients, both in the Delirium and Control groups, spent in the hallway (Figure 2). Compared to the Control group, patients identified with delirium had a longer ED LOS (median of 11.94 hours v. 8.12 hours, *P*<0.001), and had more ED room transfers (median of 5 vs 4, *P*<0.001) (Table 4). Patients identified with delirium vs those in the Control group had a significantly longer median hospital LOS (5.0 days vs 4.6 days, *P*<0.001). Patients identified with delirium had significantly higher CCI scores (*P*<0.001) (Table 2). In a multivariable logistic regression model controlling for age, race, CCI, number of room transfers, and ED LOS, the relative odds of a patient being identified with delirium increased by 3.31 times for each percent increase in hallway time (95% CI, 2.85, 3.83) (Table 5).

## DISCUSSION

While the ED is associated with an uncomfortable and potentially deliriogenic environment, there has been a dearth of studies evaluating delirium development in this unique setting and, specifically, the ED hallway.^30^,^31^ In this study we aimed to assess the association of delirium development with time spent in the ED hallway and to determine the hospital location of delirium development. Overall, we found that greater percentage of time in the ED hallway, having more ED room transfers, longer ED LOS, and increased hospital LOS were associated with delirium development. We also found that the majority of patients first developed delirium in the ED rather than on the general wards. Overall, our ED serves an older population, represented by the median age of both the Delirium and Control groups (70 and 71, respectively). In our study, patients under the age of 65 were not excluded as the study sought to identify the roles the ED hallway and environment held in impacting delirium development in all patients. Considering that delirium affects the majority of hospitalized, older adults and leads to severe outcomes in these patients and that patients 65 years and older are expected to represent 25% of all ED visits by 2030,^18^ delirium recognition and management in the ED is fast becoming an important area of research.

With regard to ED exposure, our study found a significant association between delirium and ED LOS as well as between delirium and time in the ED hallway. The nature of a busy ED potentially amplifies the environmental risk factors for delirium development, which include the absence of orientation items (e.g., legible clocks,^32^,^33^ reading glasses,^32^ hearing aids)^30^; inadequate access to natural light (lack of windows)^11^,^30^ elevated noise level and increased disruptions^32^, ^34^, ^35^; and limited interactions with familiar persons^36^ (e.g., family members, caregivers). Previous studies have found that longer ED LOS was a contributing factor to the development of delirium. A minimum of 12 hours of ED exposure has been cited as a strong predictor for onset of delirium,^4^,^15^,^37^,^38^ and some studies find it may be as few as 10 hours.^38^ In a study by Émond et al, 18% of patients with a minimum of 12 hours of ED exposure developed delirium and had subsequently longer median ED and hospital LOS.^4^ In another study by Bo et al, the authors found that an ED LOS of 10 hours or more demonstrated that the risk for incident delirium approximately doubled.^38^ These findings underline the importance of understanding how the ED environment could be contributing to the development of delirium.

The ED hallway is a unique location within the ED environment, and it has become a common area to place patients because of universal overcrowding. Patients are placed in the ED hallway as they wait for treatment beds to open or for boarding, waiting for an inpatient bed, such as a geriatric bed or bed with enhanced observation, to become available. A previous study by Han et al found that patients were even excluded from delirium assessments if they were in the hallway because of the high level of ambient noise that would impede cognitive assessments and psychiatric evaluations.^21^ If the ED hallway is preventing the standard of care from being met, then placing patients in the hallway places them at a dangerous risk for the development of delirium. For patients with baseline cognitive impairment such as dementia who are not able to advocate for their mental state without the presence of a caregiver, the ED hallway could put them at risk for even greater harm for delirium.^8^, ^20^, ^39^ To our knowledge, this is one of the first studies to evaluate the association between time in the ED hallway and delirium development. Indeed, the ED hallway stands out as an important and novel ED environmental risk factor for delirium development.

In addition to longer ED LOS and time spent in the ED hallway, we found that more ED room transfers were associated with delirium. This association has been shown in literature from other inpatient settings, where more frequent room transfers on internal medicine and geriatric medicine units contributed to delirium development.^5^, ^30^ The frequent room transfers would further harm a person’s ability to orient within their changing environment.^5^ This finding furthers the need to ensure stable hospital environments for patients at risk of developing delirium.

To improve the prevention, identification, and management of delirium in the ED and the ED hallway, providers will have to address current barriers to delirium screening. While hypoactive delirium composes upward of 70% of delirium cases,^40^–^43^ a previous study found a seven-fold risk of under-recognition.^44^ Nurses tend to use behavior such as cooperation with care as an indication of cognitive function^14^, ^18^; however, hypoactive delirium presents with psychomotor slowing and passive presentation, which causes these symptoms to be overlooked and not identified as symptoms of delirium.^34^,^35^ In this study, delirium was identified if it was treated with pharmacologic agents and special observation, which would be more characteristic for patients with symptoms of hyperactive delirium (e.g., psychomotor agitation, aggressive behavior, inappropriate behavior). This results in patients with hypoactive delirium symptoms being more vulnerable for reduced screening and symptom management.

In this study, only 0.4% of patients were identified with delirium based on CAM documentation with the remaining 99.6% of delirious patients identified through provider orders for delirium management including pharmacologic agents and special observation. Prior research has shown that compared to researchers, bedside clinicians miss delirium cases in up to 75%^20^,^45^ of patients, and compared to psychiatrists, emergency physicians miss 28% of delirium cases.^21^ The low usage of CAM, shown through this study, identifies an area of opportunity to use other efficient and effective screening tools for delirium. CAM is the most widely used delirium detection instrument, and it has been adapted for the ED^39^ and for family corroboration^46^ (FAM-CAM). Considering that the ED is universally overcrowded and nursing shortages can limit time committed to delirium screening, shorter validated screening tools may be preferred. Other screening tools such as the 4 A’s Test^4^,^35^,^47^ and Nursing Delirium Screening Scale^34^,^48^ have been identified as quick delirium screening tools that can be used routinely in the ED to improve screening compliance.

## LIMITATIONS

This study is not without limitations. First, because the time of delirium development was based on the order times for pharmacologic agents and special observation or time of an initial positive CAM screening, this provided only an approximate time of development and patients could have been experiencing delirium that did not require clinician management. Indeed, our study likely identified mostly hyperactive delirium and may have missed hypoactive delirium. As shown in other retrospective cohort studies, hypoactive delirium is difficult to detect through EHR documentation.^22^

The retrospective nature of this study presents its second limitation. We excluded patients with chief complaints indicating baseline-altered cognition in order to exclude patients with delirium at the time of ED arrival. This method reduced the number of patients with extant delirium, yet some patients with extant delirium could have been included considering that clinicians are known to miss delirium symptoms in patients.^20^,^21^,^45^ The methodology from this study, however, allowed for a larger sample size. Although there is no standard or validated way of retrospectively identifying delirium in the ED, we used a methodology based on those used in four previously published papers,^22^–^25^ including studies performed using our study site’s EHR,^24^,^25^ and we conducted a pilot abstraction to validate the method used to conduct the electronic data query.

Third, because this was a single-center study it could limit the generalizability of our results. Other EDs may have unique factors that contribute to the development of delirium that may not be reflected in our results. The methodology used for this study, therefore, would have to be validated to be applicable to other institutions.

Fourth, because the reason for placement in a hallway bed is not standardized in the ED, it is possible that patients with delirium symptoms were placed in the hallway beds to facilitate observation. Analysis of this relationship, however, indicates that the majority of the patients in the Delirium group were placed in the hallway before being identified with delirium, and delirium was identified in these patients 7.82 hours, on average, after being placed in the hallway (Supplement 1). For patients who developed delirium before being placed in the hallway, on average they developed delirium 5.06 hours before being moved to a hallway bed (Supplement 1). We interpret this to mean that hallway exposure precedes the development of delirium, playing more of a causal role in delirium development than a role in managing delirium symptoms.

The lack of standardization for bed placement and room transfers in the ED presents an underdeveloped understanding for the reason patients are placed in the hallway and transferred to different rooms. Due to ED overcrowding, this can impact the placement of patients as the volume and acuities of patients will continuously fluctuate, impacting placement of patients in a room compared to a bed in the hallway. This presents a topic to be assessed in further studies.

Additionally, to assess time spent in the ED hallway, this study depended on timestamps for room changes, including hallway bed placements. The documentation of the time of bed placement is dependent on ED staff entering these times into the patient’s EHR, which allows for variability due to potentially delayed documentation. And lastly, inconsistent and limited identification of delirium in hallway patients could be due to ED crowding or due to a direct effect of being in a hallway bed. Because we used a retrospective chart review as our method of analysis, this discrepancy is difficult to determine and would require a prospective study to understand the clinical nuances that impede the identification of delirium in the hallway. Overall, this method of identifying delirium requires further investigation.

## CONCLUSION

We found a strong association between percentage of time spent in the ED hallway and delirium development after controlling for confounding factors in a multivariable logistic regression model. Greater time spent in the ED, especially the ED hallway, increases vulnerable patients’ exposure to deleterious environmental factors identified in prior literature. Given the high rate of delirium in the ED, education and standardization of delirium prevention, screening, and management should be urgently investigated.

## Supplementary Information



## Figures and Tables

**Table 1:** Metrics used to identify delirium in electronic health record documentation.

EHR metric	Included (≥1 source of delirium management AND ≥1 key term)	Excluded
Delirium management		
Pharmacologic agents orders	Haloperidol Lorazepam Quetiapine Valproate sodium or Valproic acid	Clopidogrel Donepezil or Aricept
Observation orders	Constant observation Enhanced supervision Non-violent non-self-destructive level 1 Restraint Violent self-destructive level 2 restraint	1 to 1
CAM documentation	Positive	Negative

Key term used to document delirium symptoms	Agitation Altered mental status Cognitive decline Cognitive impairment Confusion Delirium Disorientation Encephalopathy Hallucination Memory loss Restlessness Unresponsiveness	Aggressive Alert but confused Dazed state Delusions Dementia Irritability Noncompliant Psychosis Somnolent Sun-downing

*EHR*, electronic health record; *CAM*, Confusion Assessment Method; *1 to 1*, one to one observation.

**Table 2:** Sample characteristics.

Characteristic	All patients (n = 25,675)	Control (n = 23,755)	Delirium (n = 1,920)	P-value
Age, years [median (IQR)]	71 (57, 83)	71 (57, 83)	70 (54, 85)	0.118
Gender				0.983
Female, n (%)	13,512 (52.6%)	12,502 (52.6%)	1,010 (52.6%)	
Male, n (%)	12,163 (47.4%)	11,253 (47.4%)	910 (47.4%)	
Race				<0.001
White, n (%)	15,672 (61.0%)	14,356 (60.4%)	1,316 (68.5%)	
Black, n (%)	3,850 (15.0%)	3,608 (15.2%)	242 (12.6%)	
Other/Multiracial, n (%)	3,635 (14.2%)	3,404 (14.3%)	231 (12.0%)	
Asian, n (%)	1,885 (7.3%)	1,798 (7.6%)	87 (4.5%)	
Native American/Alaska Native, n (%)	107 (0.4%)	104 (0.4%)	3 (0.2%)	
Unknown, n (%)	526 (2.1%)	485 (2.0%)	41 (2.2%)	
Ethnicity				0.891
Not Hispanic or Latino, n (%)	22,741 (88.6%)	21,036 (88.6%)	1,705 (88.8%)	
Hispanic or Latino, n (%)	2,837 (11.1%)	2,630 (11.1%)	207 (10.8%)	
Unknown, n (%)	97 (0.4%)	89 (0.4%)	8 (0.4%)	
Charlson Comorbidity Index				<0.001
0, n (%)	7,423 (28.9%)	6,891 (29.0%)	532 (27.7%)	
1, n (%)	3,844 (15.0%)	3,623 (15.3%)	221 (11.5%)	
2, n (%)	5,643 (22.0%)	5,201 (21.9%)	442 (23.0%)	
3, n (%)	2,887 (11.2%)	2,597 (10.9%)	290 (15.1%)	
4, n (%)	0 (0.0%)	0 (0.0%)	0 (0.0%)	
5, n (%)	5,878 (22.9%)	5,443 (22.9%)	435 (22.7%)	

* P-values derived from Wilcoxon rank-sum test for age and chi-square test for all other variables.

**Table 3:** Medications and orders used for delirium identification.

Orders for delirium management	n (% of Delirium group) n = 1,920
Medications	1515 (78.9%)
Lorazepam	907 (47.2%)
Quetiapine	322 (16.8%)
Haloperidol	167 (8.7%)
Valproic acid	119 (6.2%)
Bed Orders	397 (20.7%)
Constant observation	188 (9.8%)
Enhanced supervision	168 (8.8%)
Non-violent non-self-destructive level 1 restraint	38 (2.0%)
Violent self-destructive level 2 restraint	3 (0.2%)
Inpatient positive CAM screening	8 (0.4%)

*CAM*, confusion assessment method.

**Table 4:** Length of stay comparisons between the delirium and control groups.

Outcome measure	Delirium (n = 1,920)	Control (n = 23,755)	P-value
Percent hallway time [median (IQR)]	50.5% (20.6%, 77.8%)	10.8% (0.0%, 59.6%)	<0.001
ED hallway LOS, hours [median (IQR)]	5.85 (1.94, 11.53)	0.80 (0.00, 6.15)	<0.001
ED LOS, hours [median (IQR)]	11.94 (7.48, 22.04)	8.12 (5.57, 13.37)	<0.001
Number of ED room transfers [median (IQR)]	5 (4, 5)	4 (3, 5)	<0.001
Hospital LOS, days [median (IQR)]	5.0 (3.0, 8.4)	4.6 (2.8, 7.9)	<0.001

P-values derived from Wilcoxon rank-sum tests.

*ED*, emergency department; *LOS*, length of stay; *IQR*, interquartile range.

**Table 5:** Multivariable logistic regression model of the independent association between percent hallway time and development of delirium (n = 25,675).

Variable	Odds ratio	(95% CI)	P-value
Hallway time, per unit of percent change	3.31	(2.85, 3.83)	<0.001
Age, per year	0.99	(0.99, 1.00)	<0.001
Race			<0.001
White	1.00	(Reference)	
Black	0.63	(0.54, 0.73)	
Asian	0.51	(0.41, 0.65)	
Native American/Alaska Native	0.24	(0.08, 0.77)	
Other/Multiracial	0.67	(0.58, 0.78)	
Unknown	0.97	(0.70, 1.35)	
Charlson Comorbidity Index			<0.001
0	1.00	(Reference)	
1	0.91	(0.77, 1.08)	
2	1.19	(1.04, 1.36)	
3	1.62	1.38, 1.90)	
5	1.20	(1.05, 1.38)	
Total of number of room transfers, per number	1.22	(1.18, 1.26)	<0.001
ED length of stay, per hours	1.02	(1.02, 1.03)	<0.001

*CI*, confidence interval; *ED*, emergency department.
