# Supplementary material for: Increased Emergency Department Hallway Length of Stay is Associated with Development of Delirium
Source: West J Emerg Med. 2021 Apr 9;22(3):726–35. doi: 10.5811/westjem.2021.1.49320 (PMC8202999; doi:10.5811/westjem.2021.1.49320)
Supplement: Supplementary file 1 [file wjem-22-726-s001.pdf]

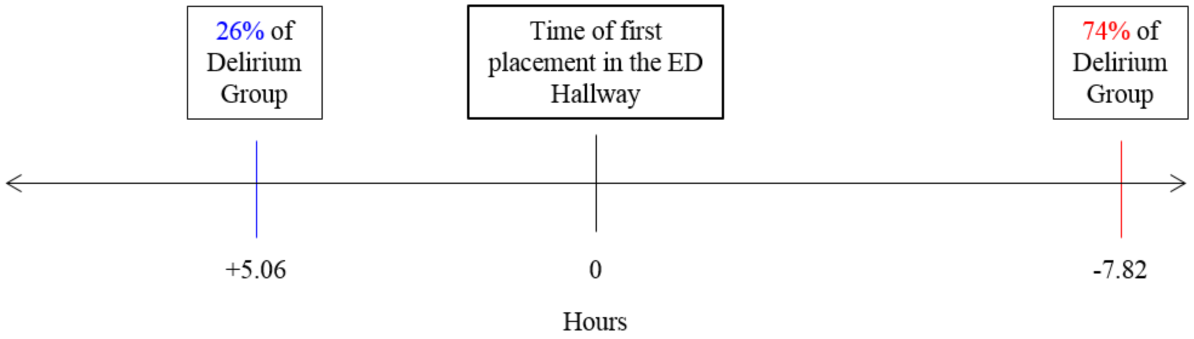

Supplement Figure. The relationship between the time of delirium development and the time of placement in the Emergency Department hallway.
ED, emergency department.
